# Monitoring variables affecting positron emission tomography measurements of cerebral blood flow in anaesthetized pigs

**DOI:** 10.1186/s13028-018-0369-5

**Published:** 2018-03-12

**Authors:** Aage Kristian Olsen Alstrup, Nora Elisabeth Zois, Mette Simonsen, Ole Lajord Munk

**Affiliations:** 1 Department of Nuclear Medicine and PET Centre, Faculty of Health, Aarhus University Hospital, Noerrebrogade 44, 10C, 8000 Aarhus C, Denmark; 2 Department of Clinical Biochemistry, Faculty of Health, Copenhagen University Hospital Rigshospitalet, Blegdamsvej 9, 2100 Copenhagen, Denmark

**Keywords:** Animal, Brain research, CBF, [^15^O]-water, Positron emission tomography, Swine

## Abstract

**Background:**

Positron emission tomography (PET) imaging of anaesthetized pig brains is a useful tool in neuroscience. Stable cerebral blood flow (CBF) is essential for PET, since variations can affect the distribution of several radiotracers. However, the effect of physiological factors regulating CBF is unresolved and therefore knowledge of optimal anaesthesia and monitoring of pigs in PET studies is sparse. The aim of this study was therefore to determine if and how physiological variables and the duration of anaesthesia affected CBF as measured by PET using [^15^O]-water in isoflurane–N_2_O anaesthetized domestic female pigs. First, we examined how physiological monitoring parameters were associated with CBF, and which parameters should be monitored and if possible kept constant, during studies where a stable CBF is important. Secondly, we examined how the duration of anaesthesia affected CBF and the monitoring parameters.

**Results:**

No significant statistical correlations were found between CBF and the nine monitoring variables. However, we found that arterial carbon dioxide tension (PaCO_2_) and body temperature were important predictors of CBF that should be observed and kept constant. In addition, we found that long-duration anaesthesia was significantly correlated with high heart rate, low arterial oxygen tension, and high body temperature, but not with CBF.

**Conclusions:**

The findings indicate that PaCO_2_ and body temperature are crucial for maintaining stable levels of CBF and thus optimizing PET imaging of molecular mechanisms in the brain of anaesthetized pigs. Therefore, as a minimum these two variables should be monitored and kept constant. Furthermore, the duration of anaesthesia should be kept constant to avoid variations in monitoring variables.

## Background

Positron emission tomography (PET) scanning of the brain of pigs is a useful tool in neuroscience [1–5]. Often, cerebral blood flow (CBF) is measured by PET scans using [^15^O]-water [5, 6]. CBF measurements are key parameters in pig studies of human conditions such as stroke [7], drug abuse [8] and brain stimulation [9]. In addition, [^15^O]-water PET scans are performed in some animal studies to assess the degree in which anaesthesia-induced variations in CBF affect the kinetic parameters of PET tracers in the brain in vivo [10]. As an example, the pharmacokinetics of the dopamine D_1_ tracer [11] C SCH23390 are affected depending on whether the pig is anaesthetized with isoflurane or propofol, which increase and decrease CBF, respectively [11]. Furthermore, CBF is tightly coupled to brain metabolism, and changes in CBF may therefore also affect results in studies of brain metabolism [12, 13]. Clearly, stable CBF is crucial in many PET studies of the brain, but little is currently known on how various physiological variables affect CBF in anaesthetized pigs.

Previously, we studied the effects of normocapnia *versus* hypercapnia on CBF in anaesthetized domestic pigs [6]. CBF increased markedly from a mean of 0.48 mL blood/min/mL brain tissue during normocapnia to 0.74 mL blood/min/mL brain tissue during hypercapnia. The result confirmed that arterial carbon dioxide tension (PaCO_2_) plays a key role in regulating CBF in pigs, and indicates that control of PaCO_2_ is a crucial factor for maintaining CBF within certain boundaries during PET studies of the brain. We needed to establish whether other physiological factors alter CBF in anaesthetized pigs in order to establish optimal experimental conditions for studying molecular mechanisms in the brain in vivo. Therefore, we carried out the present study to determine the role of arterial pH, PaCO_2_, arterial oxygen tension (PaO_2_), haematocrit (HCT), blood glucose (GLC), heart rate (HR), systolic blood pressure (SBP), diastolic blood pressure (DBP), body temperature (TEMP) and the duration of anaesthesia (TIME) on CBF in domestic pigs during isoflurane–N_2_O anaesthesia.

## Methods

### Animals

All procedures involving animals were approved by the Danish Experimental Animal Inspectorate. The present study was based on data obtained from 37 female domestic pigs (Danish Landrace x Yorkshire) weighing 38.1 ± 2.2 kg (mean ± SD). The pigs were fed a restricted pellet diet (600 g per pig; DIA plus FI, DLG, Denmark) and iron-(II)-fumarate/iron-(III)-oxide (Grynt, DLG, Denmark), and they were group housed for at least 5 days in the animal facility prior to the study. They were fasted 16 h prior to the study, but had free access to tap water. The pigs were not subjected to any specific health-monitoring program, but had no clinical signs of disease. The environmental temperature in the animal facility was 20 °C, 51% relative humidity with no specific light cycles and with the air exchanged 8 times/h.

### Anaesthesia and PET scanning

All pigs were pre-medicated with 50 mg (1.3 mg/kg) midazolam (Dormicum, Roche, Denmark) and 500 mg (13 mg/kg) ketamine (Ketalar, Pfizer, Denmark) intramuscularly. Anaesthesia was induced with 50 mg (1.3 mg/kg) midazolam and 250 mg (12.5 mg/kg) ketamine intravenously and was maintained with a vaporizer setting of 2.0% isoflurane in oxygen and N_2_O (1:2). The pigs were mechanically ventilated with a tidal volume of approximately 8 mL/kg and a frequence of 15 times/min (minute volume: 4.5 L). Heart catheters (Johnson and Johnson, Miami, FL, USA) were surgically placed in a femoral artery and a femoral vein as described in [14]. Blood gases (PaCO_2_ and PaO_2_), pH, HCT, and GLC were monitored prior to the initial [^15^O]-water PET scan, while HR, SBP, DBP, and rectal TEMP were continuously monitored using a six-channel device (Kivex, Bethesda, MD). The monitor was read immediately before the scan. Duration of anaesthesia was calculated as the time from the switching on of isoflurane and until the tracer was injected. Arterial blood samples were handled according to [15] and were analysed using an ABL 550 (Radiometer, Denmark). Isotonic saline was infused intravenously at a rate of 100–200 mL/h to prevent dehydration. After performing the baseline [^15^O]-water PET scan, the pigs were scanned with other tracers not reported here. At the end of the study, the pigs were killed with an overdose of 100 mg/kg of pentobarbitone (Veterinærapoteket, Frederiksberg, Denmark) intravenously. Necropsy of thorax (e.g. bronchopneumonia) and abdomen (e.g. peritonitis) was performed in all pigs.

### PET scan examination

The CBF was evaluated by using the radioactive PET tracer [^15^O]-water and measurements of the radioactivity as a function of time both in brain and in the bloodstream [16]. PET imaging was performed using a Siemens ECAT EXACT HR-47 tomograph (CTI/Siemens Medical Systems, Knoxville, TN Inc.). The PET camera was calibrated by a phantom containing a ^68^Ge/^68^Ga solution with known radioactivity. A 15-min transmission scan was performed before the first emission scan and was used for photon attenuation correction of the emission recordings. Dynamic PET recording was acquired in a 3D acquisition mode. The tracer [^15^O]-water was given as a 5-s intravenous injection of 500 MBq, followed by dynamic PET recordings for 5 min (20 × 3 s, 10 × 6 s, 6 × 10 s, and 6 × 20 s). Data were reconstructed using 2D iterative reconstruction (FORE OSEM). Each frame in the resulting dynamic PET image consisted of 128 × 128 × 47 voxels of 0.7 × 0.7 × 3.1 mm^3^ with a central spatial resolution of 5 mm FWHM. During the dynamic PET scans, arterial blood (7 mL/min in total 35 mL removed from the pig) was continuously sampled and radioactivity concentrations were measured every 0.5 s by an automatic blood sampler (Allogg AB, Sweden) withdrawing 7 mL blood/min, i.e. 35 mL during the PET scan. Both dynamic PET data and arterial blood data were corrected for radioactive decay measured from the start of tracer administration.

### Image analysis, kinetic modelling, and statistical analysis

A 15 mm (radius) circular formed region-of-interest (ROI) including the brain was defined in five adjacent transaxial slices of images of the mean radioactivity concentrations. The ROIs were combined to form one global volume-of-interest (VOI) containing a mixture of grey and white matter. Time courses of the radioactivity concentrations in this VOI were generated. The CBF was estimated from the dynamic [^15^O]-water PET scans. The analytical solution of the model was fitted to data in order to estimate kinetic parameters. By denoting the tissue activity concentration as *M(t)*, and the arterial blood activity concentration as *C*_*i*_*(t)*, the model predicts

1 $$M\left( t \right) = K_{1} e^{{ - k_{2} t}} \otimes C_{i} \left( t \right) + V_{0} C_{i} \left( t \right),$$

where ⊗ denotes a convolution integral. The model has three parameters: the clearance into the cell, *K*_*1*_ (mL blood/min/mL brain tissue), the reverse rate constant *k*_*2*_ (per min), and a vascular volume *V*_*0*_ (mL blood/mL brain tissue). *K*_*1*_ is assumed to be equal to CBF for freely diffusible substances such as [^15^O]-water. In practice, the blood–brain barrier limits the passage of tracer from blood to cell. This effect causes CBF to be slightly underestimated by this equation as described by the Renkin–Crone relation [17, 18]. For each data set, CBF was estimated as *K*_*1*_ by non-linear least-squares regression [19] of the one-tissue compartment model to the measured dynamic PET data. Each PET data point was weighted in proportion to the frame duration.

### Statistics

Data were examined for outliers and skewness, and tested for normality using the Shapiro–Wilk method with *P* values less than 0.05 considered significant. For normally-distributed variables, we calculated the Pearson correlation coefficient r, and the *P* value between CBF and the monitored parameters. Spearman rank order correlation method was used for variables that are not normally distributed. Correction for multiple comparisons was made using the Benjamini–Hochberg step-up procedure [20] with the critical value for false discovery rate (FDR) set to α = 0.05.

Step-wise regression with forward selection was used to test for the inclusion of best monitoring variable with threshold values for *F*-to-enter = 4.0 (minimum incremental *F* value to enter the model) and for *F*-to-leave = 3.9 (maximum incremental *F* value to remove from the model). The selection started without monitoring variables in the model. Then, we tested the inclusion of each monitoring variable that was not in the model, adding to the model the monitoring variable with the largest *F*-to-enter statistic provided that it is above the threshold. Then, it is tested to see if any variables already included have fallen below *F*-to-leave threshold. This process is repeated until no monitoring variables have *F*-statistics on the wrong side of the threshold.

## Results

CBF was calculated in 37 pigs that were PET scanned between 79 and 314 min after onset of anaesthesia. The physiological monitoring variables are shown in Table 1. For all parameters, mean values were within the porcine reference intervals [21].

**Table 1:** Estimated cerebral blood flow and measured physiological variables expressed both as mean and median

	Mean	Std dev	Median	Range	N
CBF (mL/mL/min)	0.54	0.16	0.51	0.15–0.91	37
pH	7.44	0.04	7.44	7.35–7.52	28
PaCO_2_ (kPa)	6.3	0.7	6.2	5.0–7.9	28
PaO_2_ (kPa)	18	5	16	10.7–29.0	27
HCT (%)	30	3	30	24.3–35.9	26
HR (min^−1^)	115	25	116	53–160	21
SBP (mmHg)	114	14	110	86–142	23
DBP (mmHg)	76	17	70	51–118	23
GLC (mmol/L)	4.9	1.4	4.8	2.3–8.1	26
TEMP (°C)	37.7	1.3	37.9	34.8–39.9	23
TIME (min)	115	68	127	79–314	37

*CBF* cerebral blood flow, *PaCO*_*2*_ arterial carbondioxide tension, *PaO*_*2*_ arterial oxygen tension, *HCT* haematocrit, *HR* heart rate, *SBP* systolic blood pressure, *DBP* diastolic blood pressure, *GLC* blood glucose, *TEMP* body temperature, *TIME* duration of anaesthesia, *N* number of observations

**Fig. 1:**
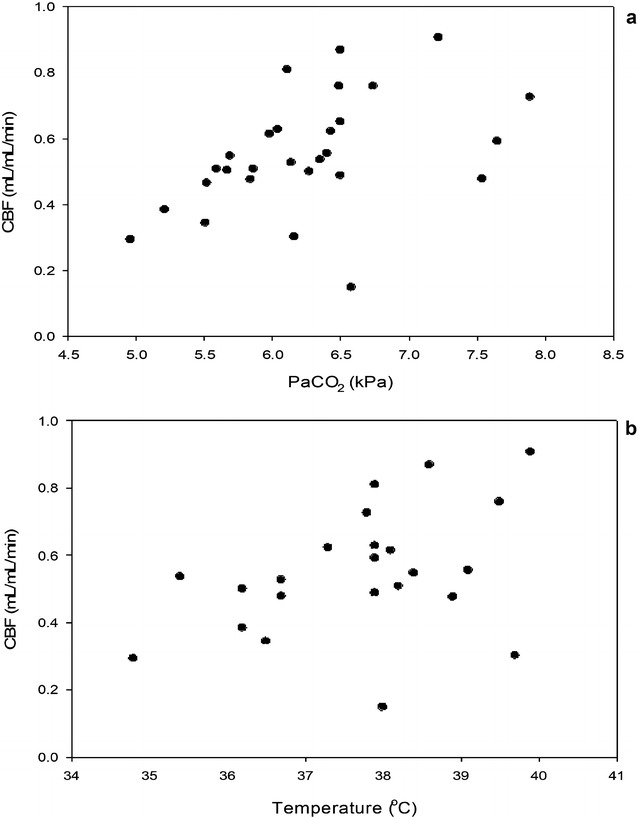
Scatter plots of **a** PaCO_2_, and **b** body temperature, the two variables that significantly contributed to prediction of cerebral blood flow

CBF and all nine monitored physiological variables passed the test for normality. The correlations and *P* values are shown in Table 2. CBF was associated with high PaCO_2_, low blood pH, high HR, and high TEMP, whereas no associations were noted between CBF and PaO_2_, HCT, SBP, DBP and GLC. However, no correlations were significant on 5% level when correcting for multiple comparisons. Thus, we screened the monitoring variables for their contribution to the prediction of CBF. Step-wise regression with forward selection was made to identify the monitoring parameters the best predicted CBF resulted in a linear combination of PaCO_2_ (P = 0.025) and TEMP (P = 0.029). No other monitoring parameters added significantly to the prediction of CBF (Fig. 1).

**Table 2:**
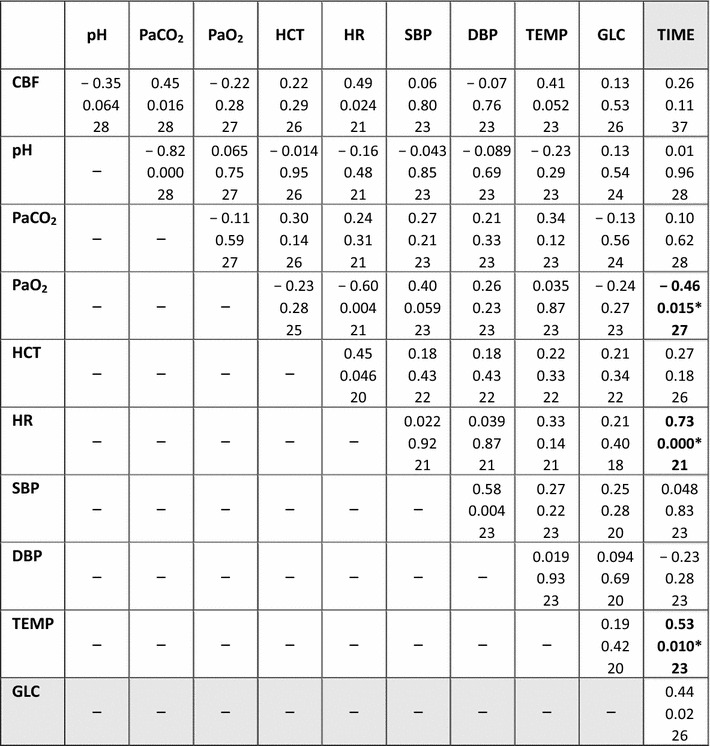
Correlation coefficients between estimated cerebral blood flow and monitoring parameters

In white background. correlation coefficients between CBF and the measured monitoring variables. Each entry contains the correlation coefficient r (Pearsons), the P value, and the number of data. Positive correlation coefficients tend to increase together, whereas inverse relationships are observed with negative correlation. In grey background, correlation coefficients between TIME and all other parameters. Each entry contains the correlation coefficient ρ (Spearman’s), the P value, and the number of data. HR, TEMP and PaO_2_ were significantly correlated to TIME after correcting for multiple comparisons. Significant correlations are shown in bold and marked with *

Finally, we examined if time after onset of anaesthesia (TIME) affected the physiological condition of the pigs by comparing TIME to all other measures. TIME did not have clear outliers and was not highly skewed but did not pass the test for normality. We therefore used Spearman’s method to test for correlations. The correlation coefficients for the relationship between TIME and CBF, and TIME and the nine monitoring variables are shown in Table 2. HR, TEMP and PaO_2_ were significantly correlated to TIME after correcting for multiple comparisons.

## Discussion

The mean CBF (0.54 mL/mL/min) was comparable with our previous study in pigs [6]. However, the variation in CBFs was high, even though standard conditions were used and the pigs were of the same sex, and had comparable age and body weight.

Out of nine monitored variables, we found significant correlations between CBF and PaCO_2_, blood pH, HR, and TEMP. However, after correction for multiple comparisons with the Benjamini–Hochberg step-up procedure, none of these monitorering variables differed significantly. The reason could be that we compared many parameters where some are highly correlated, redundant or even extraneous. Instead, the step-wise regression could identify PaCO_2_ and TEMP as the monitoring parameters that best predicted CBF. Thus, PaCO_2_ and body temperature were important predictors of CBF that should be observed and controlled. The importance of PaCO_2_ concentrations to predict CBF is in agreement with earlier studies in pigs [6, 22] and the fact that CO_2_ is a strong vasodilator in the brain. PaCO_2_ can be corrected by changing the minute volume of the respirator. We have recently shown that End-Tidal CO_2_ (ETCO_2_) can replace measurements of PaCO_2_ when there is no access to arterial blood samples in pigs, and it is therefore possible to monitor CO_2_-concentrations non-invasively [23]. The body temperature varied between slight hypothermia and normothermia. The importance of monitoring body temperature supports a previous pig study showing low CBF during severe hypothermia (body temperature < 37 °C) [24]. This effect underscores the importance of temperature monitoring and stabilization in pig brain studies. Hypothermia can be prevented by placing the pig on an electric blanket with thermostatic feedback to the temperature monitor during PET imaging procedures [25].

We found no correlations between CBF and PaO_2_, HCT, HR, SBP, DBP and GLC. It is well-known that PaO_2_ levels over 50 mmHg have no effect on CBF, while lower levels increase CBF [26]. HCT is the main determinant of blood viscosity, and in humans, higher HCT results in decreased CBF [27]. However, we found no such correlation between CBF and HCT in this study. Neither the HR, systolic nor the diastolic blood pressure was correlated to CBF. This can be explained by cerebral autoregulation which maintains a constant CBF in the mean blood pressure interval 65–140 mmHg and for variations in HR. Changes in blood pressure produce changes in cerebrovascular resistance, and this contributes to the maintenance of a constant CBF. However, a previous study reported that in ketamine anaesthetized pigs with a mean blood pressure of 102 mmHg, a 40% reduction in blood pressure (to 60 mmHg) reduced CBF with 15%, while a 43% increase in blood pressure (to 140 mmHg) increased CBF with 12% [28].

During this study some of the [^15^O]-water PET scans were delayed due to technical reasons, which caused variation of the duration of anesthesia from 79 to 314 min. Therefore, we decided to investigate if duration of anaesthesia was correlated with monitorering variables and CBF. We found that TIME was correlated with HR, TEMP and PaO_2_, but not with the other six monitorering variables and CBF. The correlation between TIME and HR can be explained by decreasing cardiac vagal activity known from dog studies (similar studies have not yet been performed in pigs) [29]. The correlation between TEMP and TIME can be explained by the fact that many pigs are slightly hypothermic shortly after anaesthesia, but body temperature is normalized by the feedback system connected to the warming blanket.

Ketamine and midazolam were used for the pre-medication and anaesthesia induction in all pigs. Isoflurane and N_2_O were used to maintain anaesthesia. While ketamine and midazolam do not seem to affect CBF, isoflurane increases CBF [30–32]. In a previous study, CBF did not increase during hypercapnia in dogs anaesthetized with 2.8% isoflurane, whereas CBF increased during 1.4% isoflurane anaesthesia [33]. In our study, the pigs were anaesthetized with a vaporisor setting of 2% isoflurane and O_2_/N_2_O (1:2) and our results indicate that in pigs, cerebral autoregulation is maintained during anaesthesia maintained with 2% isoflurane. Also N_2_O may affect CBF, as a study performed in healthy humans has shown that 30 and 60% N_2_O increase CBF compared with pure oxygen [34].

It is a limitation on the study that we have only measured global CBF, and we cannot exclude the possibility of local heterogeneities in the CBF. It is therefore possible that correlations exist between monitoring variables and specific areas of the brain not seen in the global CBF. Due to the observational nature of our study, we cannot make any conclusions about the causal relationship of the variables included. Also, several of the observed physiological variables are individually correlated which further limits conclusions about causality. Future studies should therefore aim at investigating the effects of the individual physiological variables on CBF in an interventional study, as has already been done with PaCO_2_ [6]. Until then the conclusions are drawn with caution.

## Conclusions

The results indicate that monitoring of PaCO_2_ and body temperature are crucial for maintaining stable levels of CBF and thus optimizing PET imaging of molecular mechanisms in the brain of pigs in vivo. The two variables should as far as possible be kept constant during PET scans of the pig brains. Furthermore, the duration of anaesthesia should be kept constant.
